# Enhanced RBD-Specific Antibody Responses and SARS-CoV-2-Relevant T-Cell Activity in Healthcare Workers Following Booster Vaccination

**DOI:** 10.3390/cimb46100660

**Published:** 2024-10-02

**Authors:** Lina Souan, Hikmat Abdel-Razeq, Maher A. Sughayer

**Affiliations:** 1 Department of Pathology & Laboratory Medicine, King Hussein Cancer Center, Amman 11941, Jordan; lsouan@khcc.jo; 2 Department of Medicine, King Hussein Cancer Center, Amman 11941, Jordan; habdelrazeq@khcc.jo; 3 School of Medicine, The University of Jordan, Amman 11942, Jordan

**Keywords:** COVID-19, SARS-CoV-2, immune response, vaccines, long immunity, infection, IgG levels

## Abstract

COVID-19 continues to impact healthcare workers (HCWs), making it crucial to investigate vaccine response rates. This study examined HCWs’ humoral and cellular immunological responses to COVID-19 booster dosages. We enrolled thirty-four vaccinated HCWs. Twelve received a booster. Post-immunization, the participants’ anti-COVID-19 IgG antibodies and IFN-γ secretion were assessed. The median second immunization response time was 406.5 days. Eighteen of twenty-two (81.8%) experienced breakthrough infections after the second vaccination, whereas ten out of twelve individuals who received booster doses had breakthrough infections (83.3%). Six of thirty-four HCWs (17.6%) had no breakthrough infections. Booster-injection recipients had a median antibody titer of 19,592 AU/mL, compared to 7513.55 AU/mL. HCWs with breakthrough infections exhibited a median antibody titer of 13,271.9 AU/mL, compared to 7770.65 AU/mL for those without infections. Breakthrough-infection and booster-injection groups had a slightly higher median T-cell response to antigens 1, 2, and 3. SARS-CoV-2 antibody titer and T-cell responsiveness were positively associated. HCWs sustain cellular and humoral immunity for over 10 months. Irrespective of the type of vaccine, booster injections enhance these immune responses. The results of our research are consistent with previous studies, and a multicenter investigation could validate the findings.

## 1. Introduction

The COVID-19 pandemic, triggered by the new coronavirus SARS-CoV-2 [1], has presented an unparalleled international health crisis, profoundly affecting society, economics, and healthcare systems around the globe [2]. Throughout the epidemic, healthcare workers (HCWs)—including doctors, nurses, paramedics, and support personnel—have faced unprecedented challenges. They have been at increased risk of infection since the onset of the outbreak. This has been attributed to their proximity to infected individuals, extended contact with the virus, and the direct patient-care responsibilities that frequently occur in high-risk environments like hospitals, clinics, and testing centers [3,4]. The World Health Organization predicted that there were over 80,000 COVID-related deaths among HCWs globally as of May 2021 [5]. Due to their crucial role in responding to the pandemic and the increased likelihood of being exposed to the virus, healthcare staff were given priority in vaccine programs for early immunization [6,7]. 

Vaccination is the most effective approach to prevent outbreaks, reduce morbidity, and prevent death, especially for healthcare personnel, as a result of prior influenza experiences [8]. In late 2020, COVID-19 vaccinations were introduced, changing the pandemic response. In clinical trials, Oxford-AstraZeneca (ChAdOx1-S), Pfizer-BioNTech’s (New York, NY, USA) (BNT162b2), Moderna’s mRNA-1273 (Cambridge, MA, USA), Sinovac’s CoronaVac (Beijing, China), Johnson & Johnson (New Brunswick, NJ, USA), Sputnik-V (Moscow, Russia), and Sinopharm’s (BBIBP-CorV COVID-19) (Beijing, China) vaccine were effective, driving widespread usage under emergency-use authorizations [9]. The SARS-CoV-2 spike protein is the main target of neutralizing action during viral infection [10]. Vaccination prevents symptomatic infection, hospitalization, and death by causing the production of antibodies that target this protein [11,12]. As immune responses to vaccination and clinical illness decrease over time, hosts may become more susceptible to subsequent infections [13]. However, despite the decrease in spike-protein antibody levels, vaccination may still result in detectable neutralizing antibody activity [14].

Recent breakthroughs in molecular research on COVID-19 have greatly improved our comprehension of vaccinology and the process of developing vaccines. Multiple research studies have examined different areas, ranging from investigating the molecular mechanisms of the spike glycoprotein to enhancing vaccine efficacy through genomic surveillance and other approaches. A recent example of such research is a study carried out by Matsuzaka and Yashiro that emphasized the crucial functions of the SARS-CoV-2 spike protein and examined challenges and innovative therapeutic approaches that can enhance the effectiveness of vaccines. Their research emphasized the significance of focused therapies to tackle the changing characteristics of the virus [15]. A separate investigation specifically examined the molecular processes that contribute to localized responses in a normal mRNA. A study on the COVID-19 vaccination cohort examined local temperature reactions as an objective measure of post-vaccination immunological activation. The study demonstrated a significant positive relationship between the activation of uncoupling protein 2 (UCP2) and protein kinase R-like endoplasmic reticulum kinase (PERK), which is induced by the vaccine [16]. Blankestijn et al. conducted a thorough examination of the entire blood transcriptome in long-COVID patients using unsupervised hierarchical clustering. They identified two unique groupings at the transcriptome level. Cluster 1 demonstrated an upregulation of genes related to the antiviral innate immune response, whereas Cluster 2 displayed an increase in genes linked with the adaptive immune response [17]. Moreover, it was shown that a neutralizing monoclonal antibody can mediate viral entry in coronaviruses by mimicking viral receptors, triggering conformational changes in the spike protein, and promoting entry through Fc receptor-dependent pathways, offering insights into antibody-dependent enhancement (ADE) and providing guidelines for vaccine and antiviral drug development [18].

Additional immunological investigations have demonstrated notable variations in T-cell reactions to COVID-19 based on factors such as age, gender, and comorbidities. Elderly persons frequently have diminished T-cell functions and decreased responses to immunization in comparison to younger individuals [19]. Studies have observed gender disparities in immunological responses, with males typically facing more severe consequences from COVID-19, possibly because of variations in T-cell reactions and hormonal factors [20]. Coexisting medical diseases such as HIV, diabetes, and cirrhosis have the potential to modify T-cell responses, frequently resulting in their weakening and subsequent negative consequences [21,22,23]. Patients who have weakened immune systems, such as those with cancer or who are on immunosuppressive therapy, also experience reduced T-cell immunity. This impairs their ability to adequately respond to both infections and vaccinations [24,25]. 

Furthermore, it has been demonstrated that COVID-19 mutations have significant effects on T-cell responses. The Epsilon variety, although it spreads easily, causes a weakened immune response in the host’s T-cells, possibly because of changes in the virus that impact T-cell identification [26]. However, spike-targeted immunizations have demonstrated the ability to provoke strong T-cell responses that specifically target the spike protein, even in the presence of multiple mutant strains. This suggests that these vaccines are effective in developing adaptive immunity that can identify and react to various variations [27]. This highlights the importance of vaccination strategies in maintaining effective T-cell immunity amidst the evolving landscape of SARS-CoV-2 variants.

Although the primary vaccination series provided significant protection against COVID-19 [15], mounting evidence indicated that immunity may diminish with time, especially considering the emergence of novel viral variants and the ongoing transmission dynamics [28]. Booster injections, which are supplemental doses of vaccinations provided after the original vaccination, have emerged as a deliberate strategy to overcome these challenges. Booster doses seek to prolong the duration of protection, increase neutralizing antibody titers, and strengthen cellular immunity through the reactivation and augmentation of the immune response. As a result, the risk of breakthrough infections, severe diseases, and transmission is reduced [16,17]. To compare the safety and immunogenicity of seven different COVID-19 vaccines as booster doses in individuals initially vaccinated with either the AstraZeneca (ChAdOx1 nCoV-19) or Pfizer-BioNTech (BNT162b2) vaccines, the COV-BOOST trial showed that mRNA boosters (Pfizer-BioNTech and Moderna) provided the most robust immune response, while other vaccines like AstraZeneca and Janssen also showed substantial increases in antibody levels but to a lesser extent [19]. Another comparative research evaluating the Pfizer-BioNTech and Moderna mRNA booster vaccines revealed that the boosters were well-tolerated, exhibiting mild to moderate adverse effects. Moderna generated marginally superior neutralizing antibody reactions compared to Pfizer-BioNTech boosters, particularly against different variations of the SARS-CoV-2 virus [20]. Although booster shots provide real benefits like improved immunity and protection against variants [21,23], there are issues with distribution, vaccination reluctance, and long-term effects. In addition, booster timing, composition, and dosage should be tailored to the needs of each individual. It is imperative that regional and national organizations, including dental and medical councils, continue researching the efficacy of recurrent seasonal boosting, while simultaneously advocating for the widespread use of booster immunizations [22].

Recent studies show several causes of booster-vaccine reluctance. Bakare et al. observed that Nigerian community people and primary healthcare professionals reported vaccination safety concerns, mistrust in health authorities, and misunderstanding about COVID-19 vaccines, which significantly influenced their implementation [29]. Similarly, it was found that Canadian healthcare providers and education workers were hesitant to receive both original and bivalent COVID-19 vaccines due to a perceived lack of need due to previous infections, side effects such as myocarditis, menstrual changes following COVID-19 vaccination, and unclear booster guidance [30,31,32]. In a global assessment, Kadir and Ng found that healthcare personnel’s fears about harmful effects, skepticism about vaccine efficacy over time, and insufficiently focused communication campaigns increased hesitation [33]. These findings underline the significance of safety, trust, and public-health messaging to reduce booster-vaccine reluctance.

As COVID-19 continues to influence the HCW workforce, it is crucial to investigate vaccine response rates and correlates of immunological responses. This study aimed to evaluate the kinetics at which the humoral and cellular immune responses are triggered by COVID-19 booster shots in medical staff at KHCC. The participants included those who had recently contracted COVID-19 and those who had received a third booster vaccine, either the Pfizer-BioNTech COVID-19 vaccine or the BBIBP-CorV vaccine. 

## 2. Materials and Methods

### 2.1. Study Design and Study Population

This prospective cohort study was conducted between November 2021 and July 2022 at King Hussein Cancer Center (KHCC), Jordan. The total sample size of the study was 34 HCWs, all of whom had received at least two doses of the COVID-19 vaccine. Among these participants, some experienced breakthrough COVID-19 infections between 9 days and 22 months before sampling. All participants filled out and signed a consent form approved by the KHCC-Institutional Review Board (IRB). 

The electronic medical records from the Jordanian Ministry of Health have verified the incidence of a COVID-19 infection and the specific type of immunization received. The cellular and humoral immunity of each participant was evaluated between 5 and 19 months after receiving the final vaccine dose. 

### 2.2. Quantitative Anti-COVID-19 IgG Antibody Detection

The participants’ venous blood samples were drawn only once to assess both the humoral and cellular immune responses. However, it is important to note that the participants were at varying time points from their most recent COVID-19 vaccination, whether it was their second or third dose. Each sample was five milliliters in volume. After centrifuging the samples for fifteen minutes at a relative centrifugal force (rcf) of 4300, the serum concentration was determined. The samples were stored at −80 degrees Celsius until the test was carried out. On the day of the test, the samples were gradually defrosted by first being stored in the refrigerator at a temperature of 4 degrees Celsius for twenty-four hours and then being allowed to come to room temperature for a minimum of one hour before the test. Next, the samples were examined with the SARS-CoV-2 IgG II Quant assay (Abbott Architect SARS-CoV-2 IgG). This assay is a chemiluminescent microparticle immunoassay (CMIA) designed to quantitatively assess IgG antibodies to SARS-CoV-2. It specifically targets neutralizing antibodies that bind to the receptor-binding domain (RBD) of the S1 subunit of the spike protein. The test was conducted using an Architect i1000 analyzer, manufactured by Abbott in Chicago, IL, USA, following the instructions provided by the manufacturer. The test measures a range of 21 to 40,000 AU/mL, with a positive result indicated by a value of ≥50 AU/mL. The test shows 100% agreement with the plaque-reduction neutralization tests (PRNT) [24]. 

### 2.3. Quantitation of IFN-γ-Release Assay

An interferon-gamma (IFN-γ)- release assay (Quanti-FERON^®^ SARS-CoV-2 RUO (QIAGEN)) evaluated the cellular immune response. The test comprises a series of blood collection tubes coated with one of three antigens. The SARS-CoV-2 Ag1, SARS-CoV-2 Ag2 and SARS-CoV-2 Ag3 utilize a mixture of antigens that are particular to SARS-CoV-2. These antigens stimulated lymphocytes in heparin tubes to test cell-mediated immunity (QuantiFERON SARS-CoV-2 Research Use Only; QIAGEN, Hilden, Germany). The SARS-CoV-2 Ag1 tube, as stated by the manufacturer, consists of CD4+ T-cell epitopes obtained from the receptor-binding domain (RBD) of the spike protein’s S1 subunit. The Ag2 tube contains T-cell epitopes from the CD4+ and CD8+ cells, namely from the S1 and S2 subunits of the S protein (Ag2). On the other hand, the Ag3 tube consists of T-cell epitopes from CD4+ and CD8+ cells, derived from both S1 and S2, as well as immunodominant CD8+ epitopes obtained from the entire SARS-CoV-2 genome (Ag3). Nil and mitogen tests were conducted using blood tubes, which served as negative and positive controls. The specimens were treated following the guidelines provided by the manufacturer [25,26]. IFN-γ was detected using QuantiFERON ELISA in plasma from the stimulated samples. Following a 16–24 h 37 °C incubation period, each tube was centrifuged for 15 min at 2500× *g*. Before analysis, the supernatant was transferred to a new tube and stored at −80 °C. The same procedure was used to defrost frozen serum samples. IFN-γ levels were determined by the enzyme-linked immunosorbent technique (ELISA) as directed by the manufacturer. We subtracted the nil value from the raw data to obtain the final IFN-γ values (IU/mL) for mitogen, CD4+, CD4+, and CD8+.

### 2.4. Statistical Analysis

Version 28 of the SPSS-IBM program was used for data presentation and quantitative method comparison (IBM SPSS Statistics, Armonk, NY, USA). The Mann–Whitney U-test was employed to examine the IgG antibody titer response and T-cell response to COVID-19 infection and vaccine-booster dose because the data were not normally distributed. An antibody titer was correlated with the three COVID-19 antigens using Spearman rank correlation. A *p*-value less than 0.05 is regarded as statistically significant.

## 3. Results

This study included thirty-four KHCC medical staff workers. The majority were female participants, twenty in total (58.8%), while fourteen (41.2%) were males with a median age of 37.8 years old. Twenty participants (58.8%) took the BNT162b2 vaccine knowing that all employees took the same type of vaccine for their first and second dose vaccination as per the Jordanian Ministry of Health instructions. Nine (26.5%) participants took the BBIBP-CorV COVID-19 vaccine while the remaining five (14.7%) medical staff workers took the ChAdOx1-S vaccine. Among the thirty-four HCWs, twelve (35.3%) received a booster shot, namely the BNT162b2 vaccine, regardless of their initial vaccination with a median time of 8.55 months from the sampling date. The remaining twenty-two (64.7%) did not receive a booster shot. Concerning the COVID-19 breakthrough infection, eighteen of twenty-two (81.8%) experienced breakthrough infections after the second vaccination, with a median response time of 406.5 days from the second dose, while a breakthrough infection occurred in ten out of twelve (83.3%) who had received supplemental doses. The median time between the second breakthrough infection and the booster shot was 198.5 days. A total of six healthcare workers out of thirty-four participants (17.6%) did not experience any breakthrough infections following the administration of both the second immunization shot and the booster injection (Table 1).

## 4. Effect of Vaccine-Booster Shot on Anti-COVID-19 Antibody Titer

**Figure 1:**
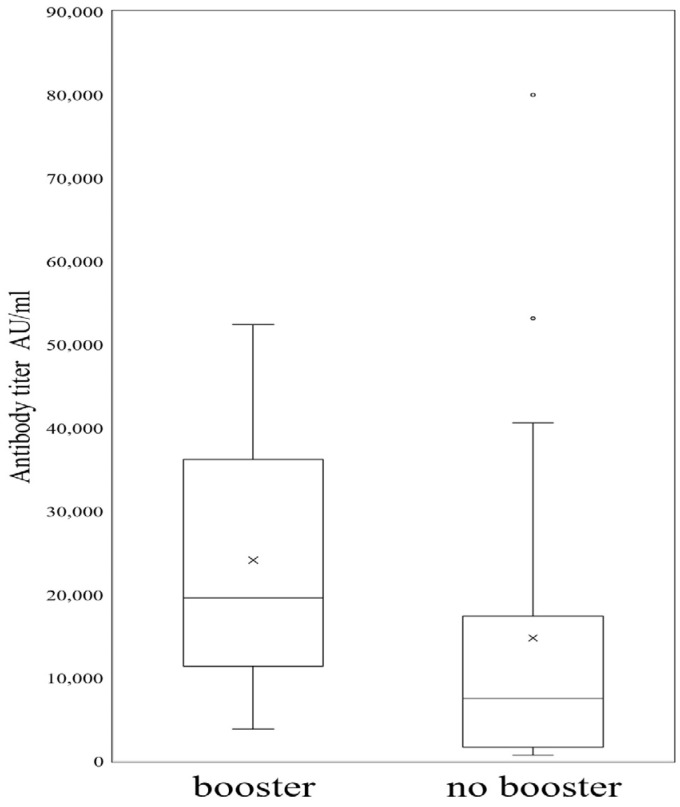
Comparison of median anti-COVID-19 IgG antibody titers in healthcare workers who got booster shots compared to those who did not.

The Mann–Whitney U-test was used to investigate the median differences in antibody response between medical staff who took BNT162b2 booster shots and those who did not take a booster shot. The results in Figure 1 show that the antibody response was significantly higher among those who took the BNT162b2 booster shot (n = 12) with a median titer of 19,592 AU/mL compared to those who did not receive the booster shot (n = 22), with a median titer of 7513.55 AU/mL, *p*-value = 0.023. 

### 4.1. Effect of Breakthrough Infection on Anti-COVID-19 Antibody Titer

The Mann–Whitney test was employed to investigate the impact of the breakthrough infection on the increase in antibody titer in healthcare workers. Our data showed that there were no significant differences between the median antibody-titer response in HCWs who had breakthrough infection (n = 28) with a median titer of 13,271.9 AU/mL compared to HCWs who did not have breakthrough infection where the median antibody titer was 7770.65 AU/mL (*p*-value = 0.23014).

### 4.2. Impact of Booster Vaccination on T-Cell Response to SARS-CoV-2 Antigens

**Figure 2:**
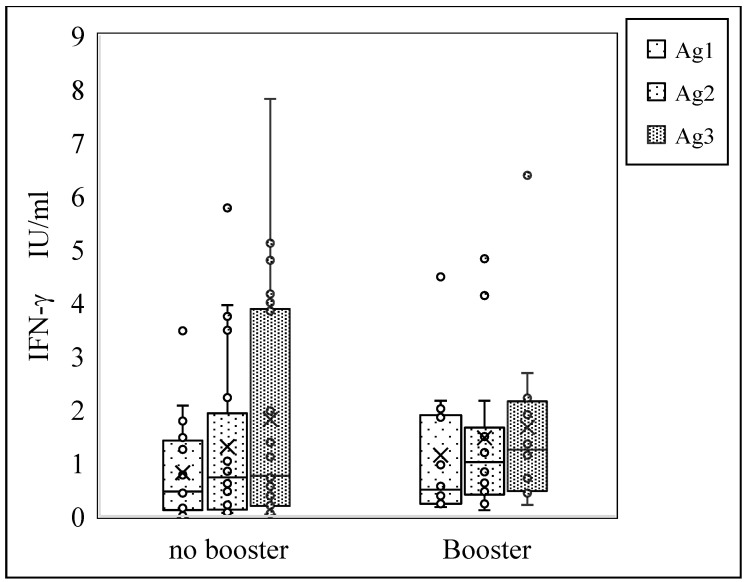
Median difference in T-cell response to SARS-CoV-2 antigens following booster immunization.

The Mann–Whitney U-test was performed to determine whether there were significant changes in the median T-cell response to SARS-CoV-2 antigens 1, 2, and 3 according to booster-dose status. The participants were divided into two groups based on their booster-vaccination status: those who did not receive a booster shot (n = 22) and those who did (n = 12). Among individuals without booster vaccination, the median differences in T-cell response were 0.46 IU/mL for Ag1, 0.725 IU/mL for Ag2, and 0.755 IU/mL for Ag3. In contrast, among those who received a booster shot, the median differences were slightly higher, with values of 0.495 IU/mL for Ag1, 1.01 IU/mL for Ag2, and 1.245 IU/mL for Ag3. The calculated X^2^ values were 107.5 for Ag1, 106.0 for Ag2, and 116.5 for Ag3, indicating substantial variability in T-cell response between the two groups across all antigens. However, the corresponding *p*-values for each antigen did not reach statistical significance, with values of 0.377 for Ag1, 0.349 for Ag2, and 0.576 for Ag3. Figure 2 shows that the median differences were not statistically significant depending on whether or not medical staff received the booster shot, *p*-value > 0.05

### 4.3. Effect of Breakthrough Infection on T-Cell Response to SARS-CoV-2 Antigens

The data revealed that HCWs who got the booster vaccine had a modest increase in the median T-cell response to antigens 1, 2 and 3 compared to HCWs who did not have breakthrough infections and the ones who did not take booster shots. The one-way analysis of variance (ANOVA) test was employed to determine whether the breakthrough infection was responsible for the modest increase in T-cell response to SARS-CoV-2 antigens. The analysis showed that the difference in the T-cell response did not reach the level of statistical significance (*p*-value > 0.05) (Table 2). Moreover, there was no significant difference in the T-cell response to the different SARS-CoV-2 antigens among the three HCW groups: the f-ratio value was 1.39628 and the *p*-value was 0.26265 for Ag1, the f-ratio value was 1.51957 and the *p*-value was 0.23 for Ag2, while for Ag3 the f-ratio value was 1.28 and the *p*-value was 0.29.

### 4.4. Investigating Correlation between Anti-COVID-19 IgG Antibody Titer and T-Cell Response to SARS-CoV-2 Antigens

A Spearman rank correlation statistical test was performed on 34 participants to explore the correlation between anti-COVID-19 IgG antibody titer and T-cell response to SARS-CoV-2 antigens. The results showed a significant positive correlation between antibody titer and T-cell response to antigens Ag1, Ag2, and Ag3 (Table 3).

## 5. Discussion

The dynamic and multifaceted nature of the humoral and cellular immune responses to COVID-19 booster injections in HCWs necessitates a thorough investigation. By understanding the complexities of these immune responses, we can gain vital insights into the effectiveness of vaccination, the durability of immunity, and the consequences of occupational health and public health measures. This understanding is crucial for guiding evidence-based policies and interventions to mitigate the impact of COVID-19 on medical staff and the broader community.

This study assessed the rate at which the humoral and cellular immune responses are activated by COVID-19 booster shots in medical staff at KHCC. The subjects consisted of individuals who had recently acquired COVID-19 and received a third dose of the vaccination as a booster. It is crucial to mention that despite the knowledge at the time of the study about the significance of taking the booster shot to enhance the immune response to COVID-19, HCWs still showed reluctance to receive the booster injection similar to what we published earlier [27]. This reluctance was one of the reasons that led to the small sample size for the study. 

When we investigated the impact of the COVID-19 booster shot on the longevity of the anti-COVID-19 antibody titer concentration, our data revealed that HCWs who received booster shots had much higher antibody titer, indicating that a booster is required to enhance humoral immune response in HCWs. This result supported other studies in normal individuals and patients with an inborn error of immunity as well as HCWs [34,35,36].

Our search into the impact of breakthrough infection on anti-COVID-19 antibody levels revealed that HCWs who experienced a breakthrough infection showed a slight increase in antibody levels. However, this increase was not statistically significant, likely due to the limited number of participants in the study. These data support the results of a recent publication by Hönning et al. where they demonstrated that heterologous COVID-19 vaccinations with mRNA-1273 boosters produced the highest IgG antibody levels [37]. 

In this study, we employed the QuantiFERON SARS-CoV-2 assay due to its reported adaptability, dependability, and accuracy in assessing cellular immune responses, as reported in numerous studies [38,39]. Our data showed that even though there was a slight increase in the median T-cell response to antigens 1, 2, and 3 in healthcare workers who got the booster vaccine, this increase did not reach the level of statistical significance (*p*-value > 0.05) due to the small sample size. 

Overall, while there appears to be a trend towards a higher T-cell response following booster vaccination, the observed differences were not statistically significant based on the available data. Further research with larger sample sizes may be needed to elucidate the impact of booster vaccination on T-cell immunity against SARS-CoV-2 antigens. Furthermore, these data indicate a positive effect on the T-cell response to SARS-CoV-2 antigens, suggesting that booster vaccination is beneficial, similar to previously reported studies in healthy individuals and immunocompromised patients [36,38]. The modest increase in T-cell response could also be attributed to the time elapsed since their last immunization and booster-vaccine shot.

Finally, the data presented in this study indicate a significantly positive correlation between the titer of anti-COVID-19 IgG antibodies and the T-cell response to the tested COVID-19 antigens, irrespective of the kind of vaccine, booster dose, or COVID-19 breakthrough infection. In essence, a rise in the T-cell response to SARS-CoV2 antigens results in a proportional increase in the level of anti-COVID-19 IgG antibodies. Accordingly, it can be postulated that there is a positive relationship between the generation of anti-COVID-19 IgG antibodies and the T-cell response to SARS-CoV-2 antigens. This association suggests that increased levels of IgG antibodies against COVID-19 may be observed in individuals with a more robust T-cell response. Individual immune system diversity, prior viral exposure, vaccination status, and other environmental or genetic factors may all have an impact on T-cell response and antibody generation. Although other studies have demonstrated that cellular and humoral immunity is detected for several months in vaccinated individuals and patients who have recovered from SARS-CoV-2 [39,40,41,42], this study is the first to demonstrate that HCWs maintain both cellular and humoral immune responses against the SARS-CoV2 virus for more than ten months following immunization. These immune responses are enhanced after receiving booster injections, regardless of the type of primary vaccine received. The presence of memory T-cells, which are essential for maintaining long-term immunity against COVID-19, may be responsible for this enduring memory. A robust and persistent immune response is ensured by the durable programming of these cells by SARS-CoV-2 inflammation [43]. For instance, previous studies have demonstrated that memory CD4+ T-cells that are specific to SARS-CoV-2 epitopes exhibit enduring responses across a variety of disease-severity levels, thereby contributing to long-term protection and sustained antibody levels [44]. Additionally, it was demonstrated that T-cells maintain their memory capacity, despite the potential for B-cells to lose memory, thereby providing a critical layer of long-term immunity against SARS-CoV-2 [45]. Memory T-cells are therefore essential for the protection against novel variants of COVID-19 and the maintenance of durable immunity. It is important to note that this study had some limitations, including the small sample size. One of the reasons for the small sample size was the time the study was conducted. As mentioned in the Materials and Methods Section the study was conducted during the COVID-19 pandemic, when only 35.3% of HCWs were willing to receive the booster vaccine, while 64.7% were not. Hence, further research, including longitudinal studies and controlled experiments, is needed to clarify the nature of the relationship between T-cell response and antibody titers in the setting of a SARS-CoV-2 breakthrough infection or vaccination. 

## 6. Conclusions

These data demonstrated that HCWs maintain both cellular and humoral immune responses against the SARS-CoV2 virus for more than 10 months following immunization, and these cellular and humoral immune responses to the SARS-CoV-2 virus were enhanced after receiving booster injections, independent of the type of primary vaccine received. The hybrid immunity generated from breakthrough infection and booster dosing supported earlier claims that hybrid immunity restricts IgG4 class-switching, enhances all isotypes, including IgG1 and IgG3, and promotes anti-N responses following infection [46,47] because IgG4 levels were shown to continue to rise six months after the second mRNA injection, indicating that there is minimal immune imprinting [48]. Our data further demonstrated that the specific T-cell response directed to the receptor-binding domain (RBD) of the S1 subunit of the spike protein (Ag1), the epitopes from the S1 and S2 subunits of CD4+ and CD8+ cells, known as (Ag2), as well as the T-cell epitopes from CD4+ and CD8+ cells generated from both S1 and S2 and the immunodominant CD8+ epitopes acquired from the full SARS-CoV-2 genome, referred to as (Ag3). This knowledge could potentially have a crucial impact on understanding the function of SARS-CoV-19-specific T-cells in terms of protection, immunological pathology, and the development of future vaccines against the SARS-CoV-2 virus. We believe that our data support other studies and can be used to augment immunization regimens, provide guidance for policy decisions, and contribute to ongoing efforts in combating the COVID-19 pandemic by generating knowledge and insights from relevant research.

## Figures and Tables

**Table 1:** Healthcare workers’ demographics.

Variables	Categories	n (%)
Gender	Male	14 (41.2%)
	Female	20 (58.8%)
Age	Median	37.8 years
Type of vaccine (The same vaccine type was used for the first and second doses.)	BNT162b2	20 (58.8%)
	BBIBP-CorV COVID-19	9 (26.5%)
	ChAdOx1-S	5 (14.7%)
Third dose of vaccine (booster shot)	Took booster shot	12 (35.3%)
	Did not take a booster shot	22 (64.7%)
COVID-19 breakthrough infection	After the second vaccine dose	18 (81.8%)
	After the booster shot	10 (83.3%)

**Table 2:** Effect of breakthrough infection on median T-cell response to SARS-CoV-2 antigens.

HCW Groups Divided According to Breakthrough-Infection Status (n = 34)	SARS-CoV-2 Antigens Median IFN-γ T-Cell Response		
	Ag1	Ag2	Ag3
No breakthrough infection (n = 6)	0.50	0.35	0.54
Breakthrough infection after 2nd vaccine shot (n = 18)	0.44	0.87	0.92
Breakthrough infection after booster shot (n = 10)	0.76	1.26	1.63
F-ratio value	1.396	1.52	1.28
*p*-value	0.26	0.23	0.29

**Table 3:** Correlation between anti-COVID-19 IgG antibody titer and T-cell response to SARS-CoV-2 antigens.

	Ag1	Ag2	Ag3
Antibody titer (AU/mL)	0.560 **	0.606 **	0.545 **

** *p* < 0.001.

## Data Availability

Data available on request due to restrictions, e.g., privacy or ethics.
